# St. George's Hospital

**Published:** 1889-10-12

**Authors:** 


### ST. GEORGE'S HOSPITAL.

Dr. Clifford Allbutt recalled to mind how vividly he recollected his own sensations when, as a raw student, he first commenced to attend lectures and "walk the hospitals." He was then "joyous," and we hope, although he did not confess as much, that he is "joyous" still. He told the students, "Your mistress is the art of medicine, and your freedom is her service." No doubt, in after times the students will find that if not medicine, certainly *practice* will be their master, and that the service will certainly *not* be freedom! Dr. Allbutt then became classical, likening the portals of medicine to the gate of Hercules, which led to a dissertation on the education of aspirants to medical studentship; and he does not seem to agree with Mr. Wheelhouse, who, in his address at Leeds before the British Medical Association, seemed desirous of reintroducing the apprenticeship system to a modified extent. Dr. Allbutt concluded a lengthy and powerful address by requiring medical men to raise their eyes to that lofty conception of duty which "Hippocrates set forth in the first chapter of his scriptures."

